# From Fragmentation to Recovery: Hydropower Impacts on River Connectivity and Fish Diversity Conservation in China’s Dongjiang River

**DOI:** 10.3390/ani15182708

**Published:** 2025-09-16

**Authors:** Huifeng Li, Yuefei Li, Lin Wang, Kun Cao, Shuli Zhu, Jinghua Luo, Jie Li, Xin Su

**Affiliations:** 1 Pearl River Fisheries Research Institute, Chinese Academy of Fishery Sciences, Guangzhou 510380, China; 2018213001@njau.edu.cn (H.L.);; 2 Key Laboratory of Aquatic Animal Immune Technology of Guangdong Province, Guangzhou 510380, China; 3 Guangzhou Scientific Observing and Experimental Station of National Fisheries Resources and Environment, Guangzhou 510380, China; 4 Fishery Resource and Environment Research Center, Chinese Academy of Fishery Sciences, Beijing 100141, China; 5 Scientific Observing and Experimental Station of Fishery Remote Sensing, Ministry of Agriculture and Rural Affairs, Beijing 100141, China

**Keywords:** Dongjiang river, dendritic connectivity index (DCI), fish diversity, habitat fragmentation, conservation strategies

## Abstract

This study quantified the Habitat Connectivity Index (DCI) of cascade dams in the mainstream of the Dongjiang River, revealing the non-linear relationship between dam passability (p) and connectivity restoration. Results showed that DCI increased slowly when *p* < 0.6 (with the magnitude of increase not exceeding 10.41), whereas an exponential response emerged when *p* > 0.8 (specifically, DCI rose by 16.53 as p increased from 0.8 to 0.9). Time-series analysis indicated that the number of dams increased from 3 to 16 between 1970 and 2020, which plunged the natural-state DCI (set at 100) to 9.01 (representing a 90.99% decrease); notably, 78.14% of the total connectivity loss occurred during the 2000–2010 period. Spatial heterogeneity analysis demonstrated that enhancing the passability of Jiantan Dam increased DCI by 4.68 (under the baseline condition of *p* = 0.8), whereas the same intervention on Sulei Dam only led to a 0.58 increase in DCI. This finding highlights the importance of key nodes for connectivity restoration and provides a scientific basis for prioritizing the enhancement of connectivity at such nodes in subsequent ecological governance. A 2024 fish community survey found that 84.2% of the recorded species were native (64 out of 76), while only 18.8% of the total individuals (617 individuals) were migratory; the dominant species were identified as generalist residents, including Oreochromis zillii, Cirrhinus molitorella, and Hemiculter leucisculus. This study identifies 0.8 as a critical threshold for connectivity restoration and provides a spatial decision-making framework for prioritizing the restoration of key dams.

## Simple Summary

This study measured the habitat connectivity (quantified via the Habitat Connectivity Index, DCI) and dam passability (p) in the mainstream of the Dongjiang River. It identified a non-linear relationship between p and DCI: specifically, DCI increased slowly when *p* < 0.6, whereas an exponential growth pattern emerged when *p* > 0.8. From 1970 to 2020, dams increased from 3 to 16, cutting natural DCI by 90.99% (78.14% lost 2000–2010). Key dams like Jiantan Dam mattered more—improving its passability lifted DCI by 4.68 (at *p* = 0.8), while 0.58 for Sulei Dam. A 2024 survey showed 84.2% native fish, 18.8% migratory, with generalist residents dominant. This study identifies *p* = 0.8 as a critical threshold for habitat connectivity restoration and provides a scientific basis for prioritizing the restoration of key dams. In turn, this work offers support for the development of nature-based ecological management strategies tailored to hydropower-dense river systems.

## 1. Introduction

Rivers, as the core of hydrological networks, form linearly connected ecological networks [1], and serve as critical components of socio-ecological systems, providing a wide array of ecological services for human societies [2,3]. However, with the rapid development of the economy and society, human activities have extensively altered river ecosystems, and such alterations have emerged as a key driver of the global environmental crisis [4,5]. river connectivity is defined as the degree to which matter, energy, organisms, and information flow and diffuse unobstructed within riverine networks—encompassing mainstreams, tributaries, upstream-downstream segments, lakes, reservoirs, marshes, wetlands, and estuaries [6,7]. It is widely recognized that the connectivity and integrity of river structure and function play indispensable roles in maintaining landscape ecological quality [8,9], optimizing water resource allocation [10], safeguarding aquatic habitats [11,12,13], supporting species migration (e.g., fish) [14], and facilitating ecological succession and genetic exchange [15,16,17]. For freshwater wildlife—particularly migratory fish—longitudinal connectivity is a decisive factor for survival, reproduction, and genetic health, making its preservation a cornerstone of aquatic biodiversity conservation.

River connectivity can be categorized into four types: longitudinal (source to estuary), lateral (channel to floodplain), vertical (surface to groundwater), and temporal (seasonal variation) [18]. Among these, longitudinal connectivity has received extensive attention due to its vital functions in maintaining species migration, ecological succession, genetic exchange, and the flow of matter and energy throughout river ecosystems [1,6,19]. Although hydropower is considered a renewable and low-carbon energy source, cascade hydropower stations can alter downstream flow regimes and physicochemical conditions, disrupt ecological structures and functions, modify regional climates and river ecosystems, and form habitat islands of varying degrees in reservoirs upstream of dams and river fragments downstream. These changes are regarded as key factors causing river habitat fragmentation and loss of longitudinal connectivity [14,20]. The habitat fragmentation caused by cascade hydropower stations is a critical issue affecting global freshwater ecosystems. Severe habitat fragmentation may lead to the formation of completely isolated metapopulations in terms of river longitudinal connectivity, and is one of the two major drivers causing the global freshwater biodiversity crisis [4,20,21].

With the advancement of ecological civilization construction in China, the loss of river longitudinal connectivity driven by cascade hydropower development has increasingly become a key constraint on the progress of ecological civilization in river basins. Globally, the impacts of cascade hydropower development on river ecosystems have become a research hotspot, and Dongjiang River Basin is no exception. In Dongjiang River Basin, the rapid socio-economic development of the Pearl River Delta region over the past 20 years has driven an unprecedented scale of water conservancy projects and water resource exploitation. However, intensive hydropower development has placed immense pressure on these populations, mirroring global threats to freshwater biodiversity. Among the 14 cascade hydropower stations below the Fengshuba Reservoir on the mainstream, 12 have been successively constructed and commissioned. Meanwhile, numerous large-scale water diversion projects, river embankments, sluice gates, drainage gates, medium-to-large reservoirs, river channel straightening projects, and navigation ports have been built and put into operation. The extensive construction of water conservancy projects and overexploitation of water resources have exacerbated river connectivity impairment. Addressing the impacts of such fragmentation on aquatic wildlife is therefore an urgent priority in the context of basin-wide ecological restoration.

In recent years, scholars worldwide have conducted systematic research on the impacts of cascade hydropower stations on river ecosystems. Internationally, He et al. [22] assessed river connectivity using pre- and post-dam biological and environmental data from the Amazon River Basin, the Colorado River in the United States, and the Conon River in the UK. They revealed that different types of dams disrupt fish migration pathways and confirmed a significant negative correlation between river fragmentation and biodiversity loss. Domestically, Cheng et al. [23] used environmental DNA (eDNA) technology to demonstrate that cascade development on the Wujiang River mainstream induced fish community homogenization, accompanied by a 45% decline in rheophilic fish species—verifying the restructuring effect of habitat fragmentation on fish assemblages. Research on the upper reaches of the Minjiang River [24] showed that the cascade hydropower station group caused a 32% proportion of river fragments, leading to the local extinction of rare species, highlighting the ecological risks of longitudinal connectivity loss.

Regarding river connectivity assessment methods, existing studies primarily focus on single barrier effects or hydrological parameter analyses. For example, research on cascade development in the Lancang River used hydrodynamic models to quantify the impact of water temperature stratification [25], but lacked a comprehensive assessment of fish migration feasibility. Although the Dendritic Connectivity Index (DCI) has been widely used in connectivity assessments in river basins such as the Amazon River [26] and the Mekong River [27,28], its application in domestic research has mostly been limited to theoretical models, with few cases of dynamic calibration combined with local fish ecological data. This study selects the DCI as the assessment indicator because it comprehensively accounts for the geometric structure and hydrological characteristics of the river networks. Its scientific advantage lies in its ability to quantify the actual impacts of river fragmentation on fish migration.

This study introduced the DCI to systematically evaluate the dynamic coupling relationship between dam passability (p) and habitat connectivity in the mainstream of the Dongjiang River across four development stages from 1970 to 2020 for the first time. By establishing a response curve between *p*-values (with a gradient of 0.1–1.0) and DCI, the threshold effect of connectivity restoration was analyzed; combined with the 2024 fish community structure data of the entire basin, the correlation mechanism between connectivity degradation and biodiversity evolution was revealed. This study aims to provide quantifiable decision-making thresholds for watershed ecological restoration, and the proposed nature-based solutions can not only meet the application needs of ecological restoration in hydropower-dense rivers but also provide a referable technical framework and practical insights for connectivity restoration and biodiversity conservation in similar watersheds.

## 2. Materials and Methods

### 2.1. Study Area

**Figure 1:**
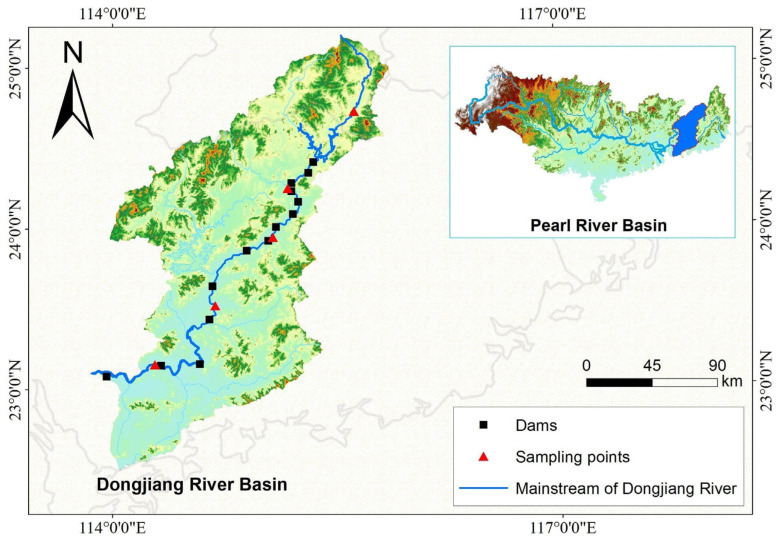
The river system map of Dongjiang River Basin.

Dongjiang river (historically named Huangshui, Xunjiang, and Longjiang) is one of the three major tributaries of the Pearl River basin. It originates from Yaji Bowl Mountain in Dongjiangyuan Village, Sanbiao Township, Xunwu County, Jiangxi Province [29]. The mainstream flows northeast to southwest through Jiangxi and Guangdong provinces, with a total length of 562 km, a total elevation drop of approximately 440 m, an average channel gradient of 0.35‰, and a drainage area of 35,340 km^2^. The long-term average annual runoff is 32.66 billion m^3^ (Figure 1). The basin is located in the subtropical monsoon climate zone, with an average annual temperature of 19–21 °C and an annual precipitation of 1500–2200 mm. Precipitation exhibits significant spatiotemporal heterogeneity: temporally, over 75% of annual precipitation occurs between April and September, which frequently induces seasonal flood and drought disasters [30]. Spatially, precipitation is higher in the upstream reaches (Jiangxi section) than in the midstream and downstream sections. Hydrological monitoring data that the average annual flow at the site of Fengshuba Reservoir is 298 m^3^/s, with the ratio of the lowest monthly flow (January) to the highest monthly flow (June) being 1:5.3, indicating a highly uneven distribution of natural runoff within the year. The upstream section in Jiangxi Province is called Xunwu River [31]. It starts to be called Dongjiang river after converging with Dingnan River in Longchuan County, Guangdong Province, and finally flows into the Pearl River’s Lion Ocean through the north and south waterways at Shilong Town, Dongguan [32,33]. For this study, the mainstream of the Dongjiang River (from the confluence of Xunwu River and Dingnan River to the estuary) was selected as the key assessment reach. This segment has a total length of 435 km, accounts for 85% of the basin’s total runoff of the basin and concentrates 90% of the basin’s water conservancy projects, including one large-scale reservoir (Fengshuba) and 12 low-head run-of-river hydropower stations. Due to these characteristics, the habitat fragmentation effects and associated ecological responses in this reach are highly typical and representative of the entire basin.

### 2.2. Methods

#### 2.2.1. Cascade Hydropower Stations Surveys

**Figure 2:**
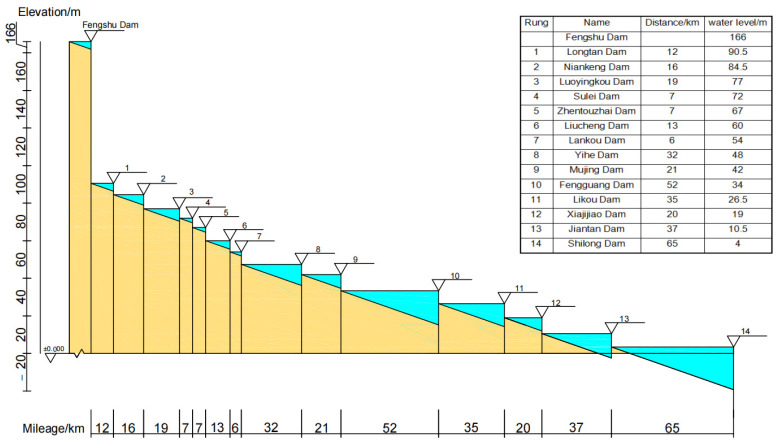
Section diagram of cascade dams in the main stream of Dongjiang river.

Small-scale hydropower stations upstream of Fengshuba Reservoir on the Dongjiang River mainstream (e.g., the Biaoxia, Changtanfeng, Shizifeng, Nanlong, Douyan, and Dutian River stations) were excluded from this study (Figure 2). In November 2023, field surveys were conducted on cascade hydropower stations; their associated fish passes in the river reach downstream of Fengshuba Reservoir, covering Heyuan and Huizhou cities in Guangdong Province. Structured questionnaires were administered to collect data on the construction timelines and operational status of these cascade hydropower stations and fish passes. The key parameters (eigenvalues) of completed and planned cascade dams along the Dongjiang River mainstream are presented in Supplementary Table S1.

#### 2.2.2. River Longitudinal Connectivity Assessment

Common quantitative calculation methods for river connectivity mainly include index method, bottleneck method, graph theory method, and hydrological modeling method [34]. For this study, the Dendritic Connectivity Index (DCI)—a key metric within the index method—was selected to assess the river longitudinal connectivity in the reaches downstream of the Fengshuba Reservoir on the mainstream of Dongjiang river.

The DCI quantifies the probability of fish movements between any two points or segments within a river network, which mainly depends on the number of cascade hydropower stations between any two points in the evaluation water area, the fish passability, and the river section length. Passability refers to the ability of fish to traverse cascade hydropower stations in both upstream and downstream directions, and river section refers to the river fragment caused by the segmentation of cascade hydropower stations [35]. Since the proportion of the evaluation river section length occupied by cascade hydropower stations themselves is extremely small, and the boundary effect generated by them is ignored, it will not affect the length of the evaluation river section [36]. At the same time, it is assumed that the ability of fish to pass through each cascade hydropower station is independent and not affected by other factors, and the cascade hydropower station blocks the internal river section is completely connected. Therefore, the longitudinal connectivity of the evaluation river section can be understood as the sum of connectivity between any two river sections [37]. The larger the DCI value, the lower the degree of river longitudinal connectivity obstruction, the lower the degree of fragmentation, the more unobstructed the fish can swim upstream and downstream, and the more conducive it is to fish survival. On the contrary, the less suitable it is for fish survival.

The mathematical calculation formula of DCI is as follows:

(1) $$DCI=\sum_{i=1}^{n}\sum_{j=1}^{n}C_{ij}\frac{l_{i}}{L}\frac{l_{j}}{L}\times 100$$

(2) $$C_{ij}=\prod_{m=1}^{M}P_{m}^{u}P_{m}^{d}$$

where n is the total number of river sections separated by cascade hydropower stations; li and lj are the lengths of river sections i and j, respectively; L is the total length of the evaluation river section; C_ij_ is the passability between river sections i and j; M is the number of cascade hydropower stations between river sections i and j; P_up,m_ and P_down,m_ are the probabilities of fish swimming upstream and downstream through the m-th cascade hydropower station, respectively, with a range of 0–1; P_m_ is the probability of fish passing through the m-th cascade hydropower station.

The Dendritic Connectivity Index (DCI) values were calculated using ArcGIS 10.2 in combination with data analysis tools based on Python3.7.2. The DCI ranges from 0 to 100, with higher values indicating superior river habitat connectivity. Given that this study primarily focuses on the impact of hydropower dam construction on river habitat connectivity between 1980 and 2020, the length of the river channel was assumed to remain unchanged to eliminate potential interference from this variable. Meanwhile, the factors influencing the dam passability (p) are complex and diverse [38]. To emphasize the effects of variations in the number and spatial distribution of dams on river habitat connectivity in the North Canal Basin, a simplifying assumption was adopted in the calculation process: the upstream passability (p^u^_m_) of dams was set equal to their downstream passability (p^d^_m_), denoted as p = p^u^_m_ = p^d^_m_.

#### 2.2.3. Fish Population Evolution

To systematically understand the current status, distribution, and long-term evolutionary trends of the aquatic ecosystem (with a focus on fish) in the Dongjiang River, and to comprehensively identify the impacts of dam construction on fish populations and community structure, this study integrated two complementary approaches: 2024 field fish surveys and historical literature analysis.

##### Field Fish Surveys

Two field surveys were conducted in July (wet season) and November (dry season) 2024 to cover seasonal variations in fish communities. The survey methods were established in accordance with relevant national standards and guidelines [39]. Sampling primarily employed three-layer gillnets with mesh sizes of 1.5 cm, 7 cm, and 15 cm, and taxonomic identification accuracy was ensured to the species level. In each sampling section, catches were obtained using different net types: fixed gillnets were deployed 15–30 m offshore each evening (19:00–05:00), while drift nets were used in deep-water areas during the daytime (6:00–18:00).

Fish were anesthetized using MS-222 (Sigma Aldrich Chemical Co., St. Louis, MO, USA), and the body length of each individual was measured in millimeters. Fish species under state protection in China, as well as threatened species listed in the IUCN (International Union for the Conservation of Nature and Natural Resources) Red List and CITES (Convention on International Trade in Endangered Species of Wild Fauna and Flora), were released back into the water after measurement.

Through taxonomic identification of specimens and analysis of collected data, a checklist of fish species composition was compiled. The identification was mainly based on references, published literature [40,41,42,43,44,45] and the FishBase database, all of which were consulted to verify synonymies, family and genus revisions, ensuring the accuracy of species names and their Latin nomenclature. Subsequently, indicators including body length, body weight, quantity, and total weight of various caught fish species were measured and counted, with body length accurate to millimeters (mm) and body weight accurate to grams (g). Meanwhile, the weight percentage, quantity percentage, and occurrence frequency of fish species at each survey station within the community were calculated through aggregation, and the dominant groups were identified by combining the Relative Importance Index (IRI) [46]. The mathematical calculation formula of the IRI is as follows:

IRI = (N% + W%) × F% × 10,000 (3)

where IRI is the Relative Importance Index; N% represents the quantity proportion of the species in the community; W% represents the biomass proportion of the species in the community; and F% represents the occurrence frequency of the species in the survey stations.

Fish species were classified into different categories based on IRI values: dominant species when IRI ≥ 1000; common species when 1000 > IRI ≥ 100; general species when 100 > IRI ≥ 10; and rare species when IRI < 10.

##### Historical Literature Analysis

To contextualize the findings of the 2024 survey within the broader framework of long-term ecological dynamics, a historical literature analysis was performed following the methodology detailed herein: by systematically reviewing relevant literary sources, changes in the population structure of fish resources in the mainstream of the Dongjiang River across distinct historical periods were synthesized, and the impacts of habitat fragmentation induced by cascading hydropower station construction on fish population structure and physiological behaviors were qualitatively assessed.

A systematic review of pertinent literature [47,48,49,50,51,52,53,54,55,56,57] was conducted to synthesize variations in the fish community structure of the Dongjiang River mainstream spanning different historical epochs (from the 1960s to the 2020s). Key datasets encompassed records of representative species (e.g., anadromous fish such as *Tenualosa reevesii*), population composition (categorizations including migratory versus resident species, and native versus invasive species), and habitat preferences (Supplementary Table S2).

## 3. Results

### 3.1. The Impact of Dam Passability p on DCI Values in Different Periods

**Figure 3:**
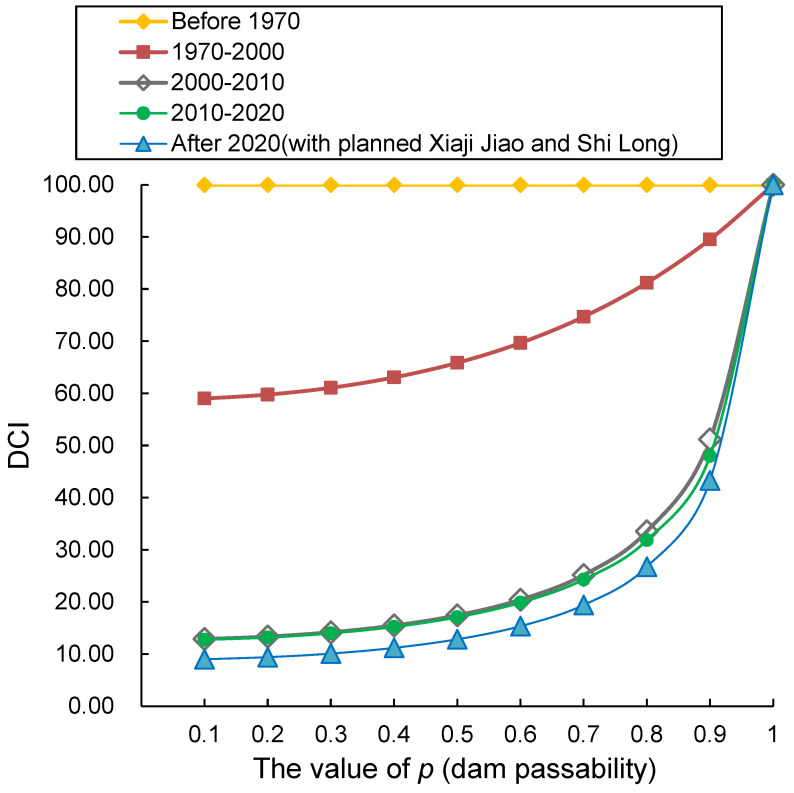
The relationship between dam passability and DCI value.

Based on the distribution of dams in the four phases, the corresponding DCI values were calculated incrementally for each phase under a gradient of dam passability (p) ranging from 0 to 1 with a step size of 0.1. As illustrated in Figure 3, DCI values generally increase with increasing p; however, the magnitude of this increase varies across phases due to differences in dam distribution (rather than “dam passability”)—a pattern reflected in the varying slopes of the p-DCI response curves. Specifically, the curve slopes remain small when 0 < *p* < 0.6, but increase sharply when 0.6 < *p* < 1.0, particularly when *p* > 0.8. Taking the post-2020 phase as an example: when p increased from 0.1 to 0.7, DCI values rose from 9.01 to 19.42 (an increase of only 10.41); in contrast, an increase in p from 0.8 to 0.9 drove DCI values to surge from 26.75 to 43.28 (an increase of 16.53). This trend indicates that low dam passability (p) constrains the effectiveness of passability improvements in enhancing river habitat connectivity—only when p is elevated to a threshold level (0.8 in this study area) can a slight increase in p trigger a significant improvement in connectivity. These findings offer critical guidance for the restoration of river habitat connectivity.

### 3.2. Effects of Changes in the Number of Dams on DCI Values

**Figure 4:**
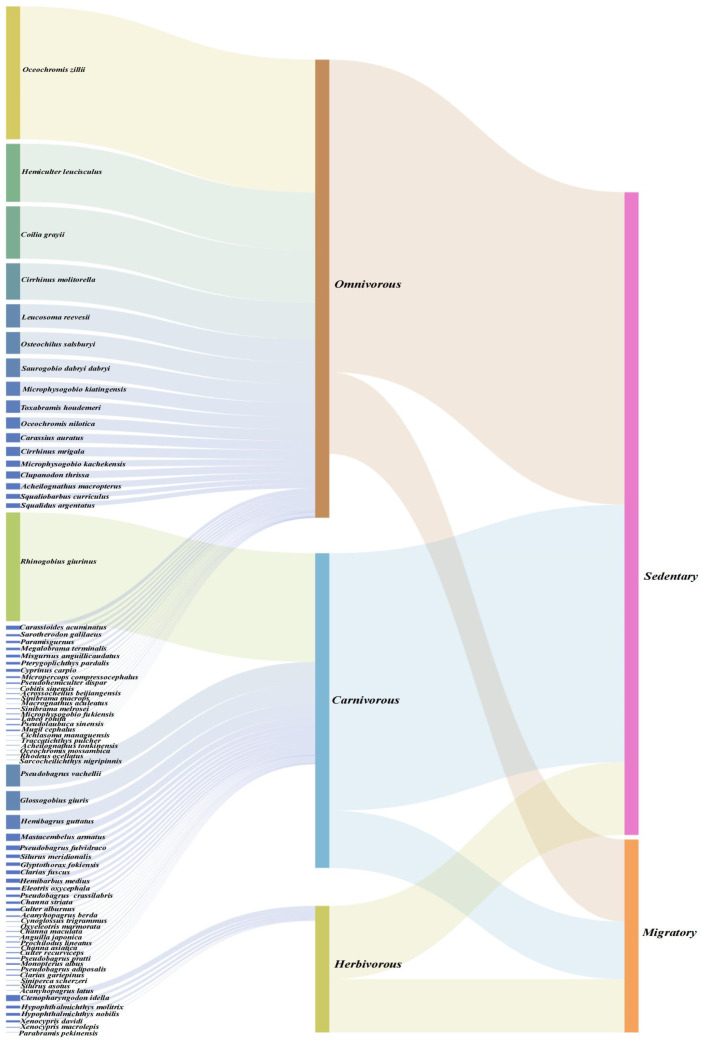
Fish Community Structure and Ecological Traits in Dongjiang River (Note: The first column lists species names, the second column indicates the feeding habits of fish, the third column shows the migration habits of fish, and high-resolution images are provided in the attached Figure S1).

From a time-series perspective, the overall river habitat connectivity decreased with the increasing number of dams constructed. As shown in Figure 4 (The relationship between dam passability and DCI value), the curve for the 1970–2000 period lies above that for 2000–2010, which in turn is above the curve for 2010–2020, and the lowest curve corresponds to the post-2020 period. Notably, when there were no dams in the basin, the river habitat connectivity value was 100, which is reflected in Figure 1 as a horizontal line parallel to the *x*-axis with an initial coordinate of (0.1, 100). After 3 dams were built during 1970–2000, the initial coordinate of the curve shifted immediately to (0.1, 59.01), meaning the river habitat connectivity value dropped to 59.01, a sharp decrease of 40.99%. During 2000–2010, 12 dams were added, and the initial coordinate of the curve became (0.1, 12.9); Accordingly, the river habitat connectivity value fell to 12.9, a decrease of 78.14% compared with the 1970–2000 period. For the 2010–2020 period, 14 dams were built, with the curve′s initial coordinate at (0.1, 12.76); There was no significant change in the river habitat connectivity value compared with 2000–2010, as evidenced by the close proximity of the two curves. After 2020, with 16 dams in total, the initial coordinate of the curve was (0.1, 9.01), and the river habitat connectivity value decreased to 9.01, a reduction of 29.39% compared with the 2010–2020 period. These results indicated that dam construction in the Dongjiang River Basin has significantly reduced river habitat connectivity, with particularly notable impacts when dams are built on natural rivers without anthropogenic disturbances.

### 3.3. Effects of Dam Passability at Different Locations on DCI Values

Taking the dam distribution in the post-2020 period as an example, this study investigated the effects of dam passability at different spatial locations on DCI values. As shown in Figure 3 (The relationship between dam passability and DCI value), the DCI value for the post-2020 period was 26.75 when dam passability (p) was 0.8. To analyze location-specific effects, the passability of each dam from Fengshu Dam to Shilong Dam was sequentially increased to 1, while maintaining the passability of all other dams at 0.8; the corresponding DCI values were calculated, and the results are presented in Table 1.

As indicated in Table 1, enhancing the passability of Jiantan Dam significantly improved the overall river habitat connectivity of the water system. In contrast, increasing the passability of Sulei Dam only raised the DCI value by 0.58, representing the lowest improvement efficiency. Jiantan Dam, located on the mainstream of the Dongjiang River, is a 21-gate structure with multiple functions, including power generation, navigation, water environment improvement, and tourism. It also serves as a key dam influencing river habitat connectivity. Due to its critical functional roles, dam removal is not a viable option; instead, to ensure habitat connectivity, it is necessary to strengthen the requirements for maintaining river habitat connectivity and implement ecological operation measures to facilitate fish passage through the dam. These results demonstrate that spatial variations in dams directly affect river habitat connectivity. For future river habitat connectivity restoration efforts, it is recommended to prioritize improving the passability of key dams (e.g., Jiantan Dam, Xiajijiao Dam, Fengguang Dam, Likou Dam, and Mujing Dam) through measures such as constructing fishways and extending gate-opening durations.

### 3.4. Composition and Ecological Traits of the Fish Community

The 2024 fish resource survey of the Dongjiang River showed that a total of 3286 fish individuals were collected, belonging to 76 species, 56 genera, 24 families, and 10 orders (Supplementary Table S3). Among these, 64 species were native, and 12 were exotic. At the order level, *Cypriniformes* exhibited the highest diversity, with 38 species accounting for 50.0% of the total, followed by *Perciformes* (16 species, 21.1%) and *Siluriformes* (12 species, 15.8%). At the family level, *Cyprinidae* ranked first with 34 species, representing 44.7% of all collected species.

Based on the Relative Importance Index (IRI) analysis presented in Section 2.2.2, the IRI values of fish in the surveyed reach are detailed in Supplementary Table S3. Specifically, there were 3 dominant species, namely *Oreochromis zillii*, *Cirrhinus molitorella*, and *Hemiculter leucisculus*; 25 common species, including *Rhinogobius giurinus*, *Ctenopharyngodon idella*, and *Carassius auratus*; 23 general species, such as *Toxabramis houdemeri*, *Sarotherodon galilaeus*, and *Mugil cephalus*; and 25 rare species, including *Acanthopagrus berda*, *Cynoglossus trigrammus*, and *Cichlasoma managuensis*.

As shown in Figure 4 (The relationship between fish ecological traits and individual abundance), the analysis of feeding habits and ecological traits of the 3286 collected fish individuals revealed the following: 1013 individuals (30.8%) were carnivorous, 2204 individuals (67.1%) were omnivorous, and 69 individuals (2.1%) were herbivorous. In terms of migration habits, 617 individuals (18.8%) were migratory, while 2669 individuals (81.2%) were resident.

Omnivorous and resident fish accounted for an absolute majority in terms of individual abundance. This characteristic indicates that the fish community in the Dongjiang River is dominated by generalist and stable-type species, reflecting that the current watershed ecosystem has developed a community structure that tends toward stability.

## 4. Discussion

### 4.1. Threshold Mechanisms in Connectivity Restoration

The non-linear response of DCI to dam passability (p) carries profound implications for habitat rehabilitation [58]. When *p* < 0.6, DCI improvements remain negligible (e.g., a mere 10.41 increase in DCI as p increases from 0.1 to 0.7 in the post-2020 period), indicating limited efficacy of engineering measures under low-passability regimes. This stems from geometric attenuation of passage success across sequential dams [59]: traversing three dams at *p* = 0.5 yields a theoretical 12.5% success rate, with actual performance further compromised by habitat fragmentation [60].

Critically, surpassing the *p* > 0.8 threshold triggers exponential DCI sensitivity (a 16.53 gain in DCI as p increases from 0.8 to 0.9). This shift occurs as “serial resistance” transforms into “conduit function”: *p* > 0.8 enables multiple passage attempts within critical migration windows, enhancing gene flow and population recruitment [61]. This aligns with fish behavioral ecology—most migratory species require above 80% per-attempt success to maintain viable populations during finite seasonal windows. Consequently, remediation should prioritize achieving *p* > 0.8 at strategic dams rather than uniform basin-wide investments.

### 4.2. Spatial Heterogeneity Dictates Restoration Efficacy

Spatial dam distribution governs connectivity restoration outcomes [62]. Taking the post-2020 phase as an example, when the baseline probability *p* = 0.8 (DCI = 26.75) was applied to the existing 16 dams (including both operational and planned structures), enhancing the navigation capacity of Jiantan Dam to 1.0 resulted in a DCI increase to 31.43 (an absolute gain of 4.68). In contrast, identical interventions at Sulei Dam only yielded a marginal DCI improvement of 0.58. This order-of-magnitude discrepancy stems from Jiantan Dam’s strategic position as a critical junction in the mainstem topological network. Its upstream reaches connect seven dam-regulated habitat patches (including Fengshu Dam), while downstream sections link eight river segments (from Shilong Dam onward), forming an ecological corridor bottleneck in the middle basin [63].

Topological analysis reveals that Jiantan Dam exerts direct influence on the connectivity of 54.3% of potential habitat patches across the entire watershed. In contrast, Sulei Dam, located downstream of tributary confluences, only associates with 12.7% of such patches, compounded by limited upstream habitat area. This finding subverts the conventional paradigm of “homogeneous restoration,” demonstrating that spatial positioning carries significantly greater weight than quantitative characteristics [64]. Historical ichthyofaunal records corroborate this conclusion: Following Jiantan Dam’s completion (2007), the proportion of migratory fish species in its upstream reaches plummeted from 35.6% (2000) to 8.9% (2010), while the concurrent downstream decline amounted to only 19.3%. Consequently, restoration efforts should prioritize topological key nodes (e.g., Jiantan, Xiajijiao, and Fengguang Dams) through fishway optimization or ecological dispatching, achieving maximum efficiency with minimal intervention.

### 4.3. Adaptive Responses of Community Structure to Connectivity Degradation

The long-term ecological impacts of longitudinal connectivity disruption on fish communities in the Dongjiang River have been dual-verified by historical literature records and the 2024 field survey. This finding aligns with the global research consensus that “hydraulic engineering induces longitudinal river fragmentation, threatening the stability of fish communities”—previous studies have highlighted that artificial barriers such as river-blocking dams and tidal sluices directly interrupt fish migration corridors, leading to the decline of migratory species resources and even their functional extinction [12,26]. Prior to the implementation of large-scale dam construction, the basin supported an anadromous fish guild, with its keystone species—*Tenualosa reevesii*—undertaking large-scale spawning migrations that extended to the upper tributaries of the Xinfengjiang River, forming the well-documented spring fishing season. Since the 1960s, the construction of tidal sluices and low-head barrages in the lower reaches has gradually impeded the upstream migration of fish; by the 1980s, juvenile fish and spawning adults of this species could still be detected in the Shilong reach of Dongguan. However, further embankment construction and the reclamation of Tonghu Lake in the 1990s nearly eliminated all records of *Tenualosa reevesii*. A retrospective analysis of the endangered causes of *Tenualosa reevesii* in the Pearl River Basin also confirmed that hydraulic engineering construction and habitat destruction are the core driving factors behind the disappearance of this species [60]. The 2024 field survey corroborated this declining trend: among the 617 collected specimens, migratory fish accounted for only 18.8%, representing a significant decrease compared to the >60% recorded before 1970. The functional extinction of *Tenualosa reevesii*, along with the concurrent disappearance of other obligate migratory species (e.g., *Coilia grayii*, *Anguilla japonica*, *Takifugu ocellatus*), is consistent with the general pattern reported in regional comprehensive studies—specifically, that anadromous/semi-anadromous fish have been replaced by sedentary, eurytopic species [15,16].

This faunal turnover is not a random phenomenon but an orderly niche reconfiguration driven by hydrological homogenization [65]. Cascading reservoirs transform lotic reaches into a series of lentic habitats by altering the original runoff regime of the river, a process that is consistent with the mechanism of “hydraulic engineering inducing river habitat homogenization and filtering fish with specific niches” [7,18]. Specifically, lentic habitats favor benthopelagic species such as *Cirrhinus molitorella*, which reproduce in slack-water nearshore zones, while disadvantaging rheophilic lithophilic fish (e.g., *Cobitidae, Gastromyzontidae*, *Gobioninae*)—the spawning substrates of these species are now buried under sediment deposited in reservoirs. Notably, sedimentation in the upstream reaches of dams and riverbed scouring in the downstream reaches are typical ecological issues caused by cascading hydropower station construction [19,23]. Meanwhile, the altered flow regime has selected fish with generalist feeding habits: omnivorous fish currently account for 67.1% of the contemporary community, which aligns with the regional trend of fish evolving toward trophic niche plasticity. A similar phenomenon has also been reported in studies on the impacts of dams in tropical rivers of southeastern Brazil [64]. *Oreochromis niloticus* is a typical representative of this shift: it can utilize algal biofilms, detrital pulses, and microinvertebrate blooms formed in eutrophic reservoirs to achieve rapid population expansion, and exhibited the highest Index of Relative Importance (IRI) in the 2024 survey. The expansion pattern of this invasive species is highly consistent with the global research conclusion that “eurytopic alien species dominate lentic environments modified by dams” [22,26].

The dominant status of such invasive species is further reinforced through an “interference-preemption cycle”: their physiological tolerance to hypoxia and temperature fluctuations endows them with a competitive advantage under the unnatural hydropeaking regimes caused by dam operation [66]. Moreover, life-history traits such as sedentary habits, a broad environmental tolerance range, and high fecundity enable them to establish self-sustaining populations, which further hinder the recolonization of migratory guilds (whose migration corridors remain disrupted). Previous studies have shown that after the loss of river connectivity, the competitive advantage of sedentary species continuously suppresses the recolonization of migratory species, forming a “pseudo-steady-state” community structure [67]. Nevertheless, the current “pseudo-steady-state” of the fish community conceals a risk of genetic erosion: small, isolated sedentary populations in fragmented habitats exhibit reduced effective population size and a significantly increased risk of inbreeding depression. Similarly, a theoretical study on *Acipenser transmontanus* populations also confirmed that river fragmentation caused by dams exacerbates the threat of inbreeding depression by reducing effective population size [17]. Integrating historical records and the 2024 survey results, it is evident that the loss of longitudinal connectivity has triggered a directional shift in the Dongjiang River fish community—from a community dominated by migratory species with high trophic diversity to one dominated by sedentary, omnivorous, and often non-native taxa. This evolutionary trajectory undermines both the functional integrity and adaptive capacity of the Dongjiang River ecosystem, which is consistent with the evolutionary pattern of “connectivity loss leading to ecosystem functional degradation” observed in large rivers such as the Yangtze River and the Mekong River [16,27,68].

### 4.4. Ecological Framework of Nature-Guided Restoration Strategy

Based on the research findings regarding the connectivity threshold effect and spatial heterogeneity in the mainstream of the Dongjiang River, the Nature-guided Restoration Strategy must adhere to three core principles: “Ecology Priority, Spatial Optimization, and Process Simulation”. Traditional engineering-based restoration often falls into the misconception of “single-point governance”—for instance, excessive investment in fishway construction at non-critical node dams while neglecting the integrity of ecological processes at the watershed scale [69]. This study proposes that restoration strategies should first focus on topologically critical nodes such as the Jiantan Dam (controlling over 50% of habitat patch connectivity) and enhance their passability above the threshold (*p* > 0.8), which can trigger a “leverage effect”. Simulation results show that when the passability (*p*-value) of three key dams (Jiantan, Xiajijiao, and Fengguang) is simultaneously increased to 0.9, the watershed-wide DCI can rise from 26.75 to 47.82, with an increase rate of 78.6%—this effect is equivalent to improving the overall *p*-value of 16 dams by 0.3. This spatial prioritization strategy not only reduces costs but also conforms to the topological energy distribution law of natural rivers—connectivity restoration at critical nodes can reactivate dormant ecological corridors [70,71].

The core of Nature-guided Restoration lies in simulating the synergistic mechanism between natural hydrological rhythms and biological migration. Historical hydrological data of the Dongjiang River show that the spawning migration of anadromous species such as *Tenualosa reevesii* is highly synchronized with the flood peaks in the flood season (April–June) [72,73,74,75]. However, the current rigid operation of sluice dams has completely disrupted this phenological correlation. It is suggested to establish a “Hydrological-Ecological Synergistic Operation System”: during critical migration windows [76] (April–June and September–October), joint operation should be implemented to generate pulsed flows downstream of key dams (pulse intensity ≥ 60% of natural flood peaks), while temporarily increasing passability to above 0.9. Practices at the Three Gorges Dam on the Yangtze River have shown that this dynamic operation has restored the natural spawning success rate of *Acipenser sinensis* to 58.7% (an increase of 32% compared with static operation), without significantly affecting power generation benefits [77]. In addition, it is necessary to construct nature-like fishways (e.g., a combination of vertical slot and pool-and-weir types) at important barriers such as the Jiantan Dam. The design of these fishways should be based on the swimming ability and rheotactic behavior parameters of target species (e.g., *Cirrhinus molitorella* and *Squaliobarbus curriculus*), rather than universal standards.

## 5. Conclusions

By coupling the DCI model with fish community data, this study systematically revealed the threshold mechanism and spatial optimization strategies for habitat connectivity restoration in the mainstream of the Dongjiang River. The core conclusions are as follows: There exists a critical threshold of 0.8 for dam passability; when passability is below this value, the overall effectiveness of connectivity restoration projects is significantly constrained. The impact of sluice dam spatial location on connectivity restoration is up to 8 times that of their quantitative characteristics, thus optimization of topologically critical nodes (e.g., Jiantan Dam) should be prioritized. The current fish community in the study area is dominated by omnivorous resident species (accounting for 81.2%), and this community structure confirms the directional shift in fish ecological strategies driven by habitat connectivity loss.

To reverse the trend of watershed biodiversity decline, this study proposes a three-stage restoration pathway and further supplements key implementation recommendations: In the first stage, the passability of critical dams (e.g., Jiantan Dam, Xiajijiao Dam) should be increased to above 0.8 to break through the critical threshold for connectivity restoration. In the second stage, an “ecological operation corridor” centered on the main channel of the mainstream should be constructed, and natural hydrological rhythms should be restored through the joint optimization of sluice dam operation modes. Meanwhile, it is suggested that the connectivity threshold (*p* > 0.8) be incorporated into the post-restoration effect evaluation system for hydropower projects in the Dongjiang River Basin, and that the identification of topologically critical nodes be regarded as a prerequisite for the approval of restoration project proposals. This research framework can provide a quantitative implementation pathway and scientific reference for “Nature-guided Restoration” in hydropower-dense rivers.

## Figures and Tables

**Table 1:** The changes in DCI after improving the passability (p) of each dam.

Cascade Dam	DCI		△DCI
	Before the Improvement of Passability p	After the Improvement of Passability p	
Fengshuba (Reservoir)	26.75	27.49	0.74
Longtan (1st Cascade)	26.75	27.45	0.7
Rengkeng (2nd Cascade)	26.75	27.43	0.68
Luoyingkou (3rd Cascade)	26.75	27.35	0.6
Suleiba (4th Cascade)	26.75	27.34	0.58
Zhutouzhai (5th Cascade)	26.75	27.38	0.63
Liucheng (6th Cascade)	26.75	27.54	0.79
Lan Kou (7th Cascade)	26.75	27.67	0.92
Yuhe (8th Cascade)	26.75	28.44	1.69
Mu Jing (9th Cascade)	26.75	28.91	2.15
Fengguang (10th Cascade)	26.75	29.59	2.84
Li Kou (11th Cascade)	26.75	29.54	2.79
Xiaji Jiao (12th Cascade)	26.75	29.59	2.84
Jian Tan (13th Cascade)	26.75	29.8	3.05
Shi Long (14th Cascade)	26.75	28.72	1.97

## Data Availability

Data is contained within the article or Supplementary Material.

## Supplementary Materials

The following supporting information can be downloaded at: https://www.mdpi.com/article/10.3390/ani15182708/s1. Table S1. The eigenvalue of completed and planned cascade dams in the maintream of East River; Table S2. The variation in fish population structure in East River based on the statistics of ecological monitoring data; Table S3. Statistics on Quantity Proportion, Weight Proportion, and Relative Importance Index (IRI) of Fish Species in the Study Area; Table S4. Fish Species and Ecological Types. Figure S1. High-resolution image of the Fish Community Structure and Ecological Traits in Dongjiang River.

## References

[1] Panagiotou A., Zogaris S., Dimitriou E., Mentzafou A., Tsihrintzis V. (2022). Anthropogenic barriers to longitudinal river connectivity in Greece: A review. Ecohydrol. Hydrobiol..

[2] Tehrani N.A., Jafary P., Sarab A.A. (2018). Ecosystem health assessment using a fuzzy spatial decision support system in Taleghan watershed before and after dam construction. Environ. Process..

[3] Wolter C., Borcherding J., Ferreira T. (2021). Characterization of European lampreys and fishes by their longitudinal and lateral distribution traits. Ecol. Indic..

[4] Reid A.J., Carlson A.K., Creed I.F., Eliason E.J., Gell P.A., Johnson P.T.J., Kidd K.A., MacCormack T.J., Olden J.D., Ormerod S.J. (2019). Emerging threats and persistent conservation challenges for freshwater biodiversity. Biol. Rev..

[5] Dudgeon D. (2019). Multiple threats imperil freshwater biodiversity in the Anthropocene. Curr. Biol..

[6] Xia J., Chen Y., Zhou Z., Zhang Q., Peng S., Wang J., Yu G.T. (2017). Review of mechanism and quantifying methods of river system connectivity. Adv. Water Sci..

[7] Kondolf G.M., Boulton A.J., O’Daniel S., Poole G.C., Rahel F.J., Stanley E.H., Wohl E., Bang A., Carlstrom J., Huber C.C.H. (2006). Process-based ecological river restoration: Visualizing three-dimensional connectivity and dynamic vectors to recover lost linkages. Ecol. Soc..

[8] Nadeau T.-L., Rains M.C. (2007). Hydrological connectivity between headwater streams and downstream waters: How science can inform policy. J. Am. Water Resour. Assoc..

[9] Pringle C. (2003). What is hydrologic connectivity and why is it ecologically important?. Hydrol. Process..

[10] Cui G., Zuo Q., Li Z., Dou M. (2012). Analysis of function and adaptability for interconnected river system network. Water Resour. Power.

[11] Gosset C., Rives J., Labonne J. (2006). Effect of habitat fragmentation on spawning migration of brown trout *Salmo trutta* L.. Ecol. Freshw. Fish.

[12] Nilsson C., Reidy C.A., Dynesius M., Revenga C. (2005). Fragmentation and flow regulation of the world’s large river systems. Science.

[13] Sun P., Wang L., Wang J., Wang C. (2016). A study of the effect of sluices and dams on river habitat connectivity. China Rural Water Hydropower.

[14] Li R., Chen Q., Duan C. (2011). Ecological hydrograph based on *Schizothorax chongi* habitat conservation in the dewatered river channel between Jinping cascaded dams. Sci. China Technol. Sci..

[15] Zheng P., Jiang X., Cao L., Wang J., Jiang Z. (2022). Long-term changes in the functional trait composition and diversity of fish assemblages in eastern plain lakes under the regime of river-lake connectivity loss. J. Lake Sci..

[16] Xie P. (2017). Biodiversity crisis in the Yangtze River: The culprit was dams, followed by overfishing. J. Lake Sci..

[17] Jager H.I., Chandler J.A., Lepla K.B., Winkle W.V. (2001). A theoretical study of river fragmentation by dams and its effects on white sturgeon populations. Environ. Biol. Fishes.

[18] Wang Q., Pang X., Li X., Wang Z., Yuan X., Zhang Y. (2019). Assessment method for the influence of hydroelectric dams on the physical habitat quality and longitudinal connectivity of rivers: A case study of the Wubu and Zaodu rivers. Acta Ecol. Sin..

[19] Lv J., Wang X., Liu W., Wang Y., Wei C., Wu J. (2017). Longitudinal connectivity and fish habitat of main tributaries in Songhuajiang River Basin. Water Resour. Prot..

[20] Oldford G., Cote D., Kehler D.G., Riefesel G.R., Wierama Y.F. (2023). FIPEX v10.4: An ArcGIS Desktop Add-in for assessing impacts of fish passage barriers and longitudinal connectivity of rivers. SoftwareX.

[21] Dudgeon D., Arthington A.H., Gessner M.O., Kawabata Z., Knowler D.J., Leveque C., Naiman R.J., Richard A.H.P., Soto D., Stiassny L.M.J. (2006). Freshwater biodiversity: Importance, threats, status and conservation challenges. Biol. Rev..

[22] He F., Zarfl C., Tockner K., Olden J.D., Campos Z., Muniz F., Svenning J.C., Jähnig S.C. (2024). Hydropower impacts on riverine biodiversity. Nat. Rev. Earth Environ..

[23] Cheng R., Zhou X., Zhang Y., Cheng R., Zhou X., Zhang Y., Li Q., Zhang J., Luo Y., Chen Q. (2024). eDNA reveals spatial homogenization of fish diversity in a mountain river affected by a reservoir cascade. J. Environ. Manag..

[24] Mu Y. (2015). The Impact of Cascade Hydropower Development on River Depletion and Dewatering—A Case Study of the Upper Minjiang River. Master’s Thesis.

[25] Chen A., Wu M., Chen K.Q., Sun Z.Y., Shen C., Wang P.Y. (2017). Main issues in environmental protection research and practice of water conservancy and hydropower projects in China. Water Sci. Eng..

[26] Barbarossa V., Schmitt R.J.P., Huijbregts M.A.J., Zarfl C., Schipper A.M. (2020). Impacts of current and future large dams on the geographic range connectivity of freshwater fish worldwide. Proc. Natl. Acad. Sci. USA.

[27] Grill G., Dallaire C.O., Chouinard E.F., Sindorf N., Lehner B. (2014). Development of new indicators to evaluate river fragmentation and flow regulation at large scales: A case study for the Mekong River Basin. Ecol. Indic..

[28] Shaad K., Souter N.J., Farrell T., Vollmer D., Regan H.M. (2018). Evaluating the sensitivity of dendritic connectivity to fish pass efficiency for the Sesan, Srepok and Sekong tributaries of the Lower Mekong. Ecol. Indic..

[29] Zeng X.H. (2021). Analysis and evaluation of water environment effect of Dongjiang river Basin regulation in dry season. Pop. Sci. Technol..

[30] Dong M.Y., Jiang Y., Li Y.P., Ren F.P. (2010). Analysis of precipitation change trend in Dongjiang river Basin over the past 46 years. Hydrology.

[31] Jiang X., Jiang Z.Y., Li Z.Y., Su J., Tang L.N., Wu M.D., Wang Y.J. (2025). A framework for the construction of effective landscape ecological network with integrating hydrological connectivity: A case study in Dongjiang River Basin, China. J. Environ. Manage..

[32] Shi J.Z., Wu W.J., Feng Z.Z. (2015). Study of cascade hydropower stations regulation on Dongjiang river based on water quality control model. Guangdong Water Resour. Hydropower.

[33] Liu J.F. (2018). Analysis of water regulation effect of Dongjiang river Basin in Guangdong Province. Guangdong Water Resour. Hydropower.

[34] Gao Y.Q., Xiao X., Ding M.M., Tang Y.Q., Chen H.Y. (2018). Evaluation of plain river network hydrologic connectivity based on improved graph theory. Water Resour. Prot..

[35] Li X.H., Wang Y.Y., Cui X.H., Jia G.D., Yu X.X. (2025). Spatiotemporal variations of hydrological connectivity and their driving factors in the Luan River Basin. Res. Soil Water Conserv..

[36] Zhang R. (2020). Connectivity Planning Research of Dujiangyan River System. Master’s Thesis.

[37] Lu J., Wang X.G., Wang Y.M., Wei C.F., Liu H.C., Zhang Z. (2017). Lateral Connectivity Analysis of River Wetlands in the Songhua River Basin. South North Water Transf. Water Sci. Technol..

[38] Ahmad H., Miranda L.E., Dunn C.G., Boudreau M.R., Colvin M.E., Dash P. (2025). Confluence of Time and Space: An Innovation for Quantifying Dynamics of Hydrologic Floodplain Connectivity with Remote Sensing and GIS. River Res. Appl..

[39] Ministry of Water Resources of the People’s Republic of China (1996). SL167-96 Specifications for the Investigation of Fishery Resources in Reservoirs.

[40] Compilation Group of the Guide to River Aquatic Biological Surveys (2014). Guide to River Aquatic Biological Surveys.

[41] Chen Y.Y. (1998). Fauna Sinica, Osteichthyes: Cypriniformes (Middle Volume).

[42] Le P.Q. (2000). Fauna Sinica, Osteichthyes: Cypriniformes (Lower Volume).

[43] Editorial Committee of Fauna Sinica (2018). Fauna Sinica, Osteichthyes: Siluriformes.

[44] Guangdong Provincial Fisheries School (1989). Freshwater Fishes of Guangdong.

[45] Pearl River Fisheries Research Institute (1989). Fishes of the Pearl River.

[46] Li X.S., Yu Z.H., Sun S., Jin X.S. (2013). Niche Breadth and Overlap of Dominant Fish Species in the Yangtze Estuary and Adjacent Waters. Chin. J. Appl. Ecol..

[47] Ye F.L., Yang P., Song B.L. (1991). The Fish Fauna of the Dongjiang River. J. Zhanjiang Fish. Coll..

[48] Gao W.F. (2010). Fish Resources and Conservation Strategies in Dongjiang (Huizhou Section). Fish. Sci. Technol..

[49] Li G.F., Zhao J., Zhu X.P., Zhang J.D., Zhao H.H., Liu L., Chen G., Chen K.C., Chen Y.L., Chu Q.Z. (2013). Investigation and Research on Freshwater Fish Resources of Guangdong.

[50] Li B.W., Lan Z.J., Li Q., Huang Y.Q., Mo J.H., Wang X.B., Li C.Z., Li M. (2011). Investigation of Fish Resources in Freshwater and Estuary of Dongguan City. South China Fish. Sci..

[51] Liu Y., Lin X.T., Sun J., Zhang P.F., Chen G.Z. (2011). Fish Community Changes in Huizhou Segment of Dongjiang River. Chin. J. Zool..

[52] Liu Y. (2011). Fish Community Changes and the Evaluating the Biotic Integrity in Dongjiang River Mainstream. Master’s Thesis.

[53] Deng F.Y., Zhang C.G., Zhao Y.H., Zhou Q.H., Zhang J. (2013). Diversity and Community Structure of the Fishes in the Headstream Region of the Dongjiang River. Chin. J. Zool..

[54] Yang Y., Wang S., Cui Y.D. (2020). Research on Water Environment and Ecology in Dongjiang River.

[55] Yang Z.P., Lu Q.Q. (2016). Exotic Fish Potential Hazards in Dongguan Reaches of Dongjiang River and Its Coping Strategies. J. Green Sci. Technol..

[56] Wang S. (2016). Structure of Fish Food Webs and Energy Flow of Ecosystems in the East River. Ph.D. Thesis.

[57] Ding Y.X. (2018). General Situation and Change Rule of Main Economic Fish Resources in Dongjiang River Basin. Master’s Thesis.

[58] Yang T., Chen Y.Q., Chen X., Yang H.W., Chen X.H., Jiang T. (2009). Hydrological Variation Caused by Damming in the Middle and Upper Reaches of the Dongjiang River in South China under Complex Environment. J. Lake Sci..

[59] Geist D.R., Brown R.S., Cullinan V., Brink S.R., Lepla K., Bates P., Chandler J.A. (2005). Movement, Swimming Speed, and Oxygen Consumption of Juvenile White Sturgeon in Response to Changing Flow, Water Temperature, and Light Level in the Snake River, Idaho. Trans. Am. Fish. Soc..

[60] Tan X.C., Guo X.G. Retrospective Analysis of the Causes of the Endangerment of *Tenualosa reevesii* in the Pearl River System. Proceedings of the 2014 Annual Academic Conference of the Fisheries Resources and Environment Branch of the Chinese Fisheries Society.

[61] Zhang B. (2017). Analysis of Discharge Integration at Dongjiang Hydrological Station. Water Power Econ..

[62] Liao G.Z. (1997). Breeding Protection of Megalobrama hoffmanni in the Pearl River. Guangdong Sci. Technol..

[63] Jalkanen J., Toivonen T., Moilanen A. (2019). Identification of Ecological Networks for Land—Use Planning with Spatial Conservation Prioritization. Landsc. Ecol..

[64] Santos A.B.I., Albieri R.J., Araujo F.G. (2013). Influences of Dams with Different Levels of River Connectivity on the Fish Community Structure along a Tropical River in Southeastern Brazil. J. Appl. Ichthyol..

[65] Yujun Y., Yanning G., Shanghong Z. (2022). The Impact of Dams on the River Connectivity of the Two Largest River Basins in China. Rivers Res. Appl..

[66] Pryke J.S., Samways M.J. (2012). Ecological Networks Act as Extensions of Protected Areas for Arthropod Biodiversity Conservation. J. Appl. Ecol..

[67] Wang G.X., Liu G.M., Chang J. (2005). Review of Ecological Hydrology Research at Basin Scale. Acta Ecol. Sin..

[68] Priyadarshana T., Asaeda T. (2007). Swimming Restricted Foraging Behavior of Two Zooplanktivorous Fishes Pseudorasbora parva and *Rasbora daniconius* (Cyprinidae) in a Simulated Structured Environment. Environ. Biol. Fishes.

[69] Ferreira L.V., Cunha D.A., Chaves P.P., Matos D.C.L., Parolin P. (2013). Impacts of Hydroelectric Dams on Alluvial Riparian Plant Communities in Eastern Brazilian Amazonian. An Acad. Bras. Cienc..

[70] Schiemer F., Keckeis H., Winkler G., Flore L. (2001). Large Rivers: The Relevance of Ecotonal Structure and Hydrological Properties for the Fish Fauna. Large Rivers.

[71] Agócsová A., Hgyeová M., Chodasová Z., Ondrejika O., Jamen U. (2020). River Restoration as a Method Towards Harmonization of Natural Habitats in the Context of Ecological Corridors Preservation: A Case Study on the Hron River. IOP Conference Series: Materials Science and Engineering.

[72] Mattocks S., Hall C., Jordaan A. (2017). Damming, Lost Connectivity, and the Historical Role of Anadromous Fish in Freshwater Ecosystem Dynamics. BioScience.

[73] Rodeles A.A., Galicia D., Miranda R. (2020). A New Method to Include Fish Biodiversity in River Connectivity Indices with Applications in Dam Impact Assessments. Ecol. Indic..

[74] Magilligan F. (2014). River Restoration by Dam Removal: Assessing Riverine Re—Connectivity Across New England.

[75] Humston R., Bernard T., Thomas M., Casto E., Barnard A., Hallacher J. (2025). Documenting Changes in Fish Passage and Ecological Connectivity Following Low-Head Dam Removal: Challenges and Opportunities in a Mid-size River. Authorea.

[76] Liu H.Z. (2024). Discussion on Several Issues Regarding the Conservation of the Chinese Sturgeon (*Acipenser sinensis*). Acta Hydrobiol. Sin..

[77] Raabe J.K., Hightower J.E. (2014). Assessing Distribution of Migratory Fishes and Connectivity following Complete and Partial Dam Removals in a North Carolina River. N. Am. J. Fish. Manag..

